# USING NETWORK ANALYSIS TO EXPLORE DIFFERENCES IN DIETARY INTAKES ACROSS OLDER ADULTHOOD

**DOI:** 10.1093/geroni/igad104.0291

**Published:** 2023-12-21

**Authors:** Kyla Shea, Gail Rogers, Kathryn Barger

**Affiliations:** Tufts University, Boston, Massachusetts, United States; Tufts University, Boston, Massachusetts, United States; Tufts University, Boston, Massachusetts, United States

## Abstract

Federal nutrition guidance in the Unites States (US) is based on the premise that healthy diets can help delay the onset or progression of age-related disease and disability. Yet, little is known about if or how dietary intakes differ across the older adulthood age span. We used NHANES 2015-2016 and 2017-March 2020 data to examine the dietary intakes of 60-69 year old (n=2079), 70-79 year old (n=1181) and 80+ year old (n=644) adults in the US. Mutual information was used to infer network relationships among all possible pairs of fourteen What We Eat in America food group categories. Based on edge weights, we observed some similarities and some differences in the manner in which food groups were co-consumed across older adulthood. For example, fruit and dairy were co-consumed similarly in the three age groups. Among the 60-69 and 70-79 year olds, protein and vegetables were co-consumed, while fruits were consumed with grains and mixed dishes. However, in the 80+ year olds, vegetables were mostly consumed with fats and oils, but less so with protein, and grains were consumed with dairy, but less so with fruit. Based on a network science approach, the manner in which food groups are consumed appears to change throughout older adulthood. Future studies are needed to evaluate consumption patterns of specific foods among age-defined and other subgroups of older adults and explore potential associations with age-related health outcomes.

